# *Leishmania infantum* arginase: biochemical characterization and inhibition by naturally occurring phenolic substances

**DOI:** 10.1080/14756366.2019.1616182

**Published:** 2019-05-24

**Authors:** Andreza R. Garcia, Danielle M. P. Oliveira, Ana Claudia F. Amaral, Jéssica B. Jesus, Ana Carolina Rennó Sodero, Alessandra M. T. Souza, Claudiu T. Supuran, Alane B. Vermelho, Igor A. Rodrigues, Anderson S. Pinheiro

**Affiliations:** a Graduate Program in Pharmaceutical Sciences, School of Pharmacy, Federal University of Rio de Janeiro, Rio de Janeiro, Brazil;; b Department of Biochemistry, Institute of Chemistry, Federal University of Rio de Janeiro, Rio de Janeiro, Brazil;; c Department of Natural Products, Farmanguinhos, FIOCRUZ, Rio de Janeiro, Brazil;; d Department of Drugs and Medicines, School of Pharmacy, Federal University of Rio de Janeiro, Rio de Janeiro, Brazil;; e Neurofarba Department, Università degli Studi di Firenze, Sezione di Scienze Farmaceutiche, Florence, Italy;; f Department of General Microbiology, Institute of Microbiology Paulo de Goes, Federal University of Rio de Janeiro, Rio de Janeiro, Brazil;; g Department of Natural Products and Food, School of Pharmacy, Federal University of Rio de Janeiro, Rio de Janeiro, Brazil

**Keywords:** *Leishmania infantum*, visceral leishmaniasis, arginase, inhibitor, rosmarinic acid, caffeic acid

## Abstract

Inhibition of *Leishmania* arginase leads to a decrease in parasite growth and infectivity and thus represents an attractive therapeutic strategy. We evaluated the inhibitory potential of selected naturally occurring phenolic substances on *Leishmania infantum* arginase (ARGLi) and investigated their antileishmanial activity *in vivo*. ARGLi exhibited a *V*_max_ of 0.28 ± 0.016 mM/min and a *K*_m_ of 5.1 ± 1.1 mM for L-arginine. The phenylpropanoids rosmarinic acid and caffeic acid (100 µM) showed percentages of inhibition of 71.48 ± 0.85% and 56.98 ± 5.51%, respectively. Moreover, rosmarinic acid and caffeic acid displayed the greatest effects against *L. infantum* with IC_50_ values of 57.3 ± 2.65 and 60.8 ± 11 μM for promastigotes, and 7.9 ± 1.7 and 21.9 ± 5.0 µM for intracellular amastigotes, respectively. Only caffeic acid significantly increased nitric oxide production by infected macrophages. Altogether, our results broaden the current spectrum of known arginase inhibitors and revealed promising drug candidates for the therapy of visceral leishmaniasis.

## Introduction

Leishmaniasis is an infectious-parasitic disease caused by different species of the genus *Leishmania* and transmitted by the female of the phlebotomine insect. Leishmaniasis is classified as a neglected tropical disease, occupying the ninth position among the most prevalent diseases in the world. It is estimated that 1.5–2 million people are infected annually, and that 350 million people live at risk of infection^1^^,^^2^. Visceral leishmaniasis (VL), or kalazar, is the most severe clinical manifestation of the disease, being responsible for high mortality rates in the absence of proper diagnosis and treatment^3^. According to WHO, in 2017, 20,792 new cases (94% of total cases) of VL occurred in seven countries: Brazil, Ethiopia, India, Kenya, Somalia, South Sudan, and Sudan^4^.

There are currently no vaccines for human VL, and treatment consists of the use of chemotherapeutic agents, such as pentavalent antimonials, amphotericin B, and miltefosine. However, those treatments show high toxicity, variable efficacy, and contribute to the emergence of resistant *Leishmania* strains^5^. Therefore, there is an urgent need for novel antileishmanial agents as well as the discovery of new therapeutic targets that may lead to a safer and more effective treatment of VL.

Arginase (E. C. 3.5.3.1, L-arginine aminohydrolase) is a metalloenzyme that catalyses the hydrolysis of L-arginine into L-ornithine and urea, participating in the urea cycle. Arginase exhibits a trimeric structure with one active site present in each monomer. Each active site contains two manganese ions, which are responsible for activating a water molecule forming a metal-hydroxide ion that attacks the guanidine carbon of L-arginine^6^. In mammals, two arginase isoforms are found, arginase I and II. They catalyse the same reaction but differ in cellular expression, regulation, and subcellular localisation^7^. In *Leishmania* species, arginase regulates parasite growth, differentiation, and infectivity^8^^,^^9^. Roberts et al. have shown that an arginase knockout mutant of *L. mexicana* is unable to grow *in vitro*. Addition of exogenous ornithine and/or the polyamine putrescine restores *L. mexicana* growth, indicating that growth arrest is probably due to the lack of these substances^8^. Moreover, putrescine is a precursor for the biosynthesis of trypanothione, which is central for parasite protection against reactive oxygen species^10^.

As a strategy for the development of safer and more effective antileishmanial drugs, several efforts have been made to find specific parasite arginase inhibitors. Previously, inhibitors of both synthetic and natural origin have been described. However, most studies have focussed on enzymes from *Leishmania* species causing the tegumentary form of the disease^11–19^. To the best of our knowledge, there is a single report on *Leishmania infantum* arginase. In this specific study, enzyme inhibition assay was performed with cellular extracts and did not employ the purified enzyme^20^. Here, we biochemically characterised recombinant arginase from *L. infantum* (herein referred as ARGLi) and evaluated its inhibition by a panel of fourteen naturally occurring phenolic substances. We used molecular docking to gain further insights into the mechanism of inhibition. In addition, we investigated the effects of these substances on parasite biology and the mammalian host cell response to *L. infantum* infection.

## Materials and methods

### Chemicals

CHES buffer (2-(Cyclohexylamino)ethanesulfonic acid), dimethyl sulfoxide, Dulbecco’s modified Eagle’s medium (DMEM), Schneider’s *Drosophila* medium, resazurin, amphotericin B, and thiazolyl blue tetrazolium bromide (MTT) were obtained from Sigma-Aldrich (St. Louis, MO). Foetal bovine serum (FBS) was purchased from LGC Biotecnologia (São José, Cotia, Brazil).

### Expression of ARGLi

The plasmid containing the gene encoding *L. infantum* arginase was commercially obtained from Genscript USA (Piscataway, USA). ARGLi was cloned into the RP1B plasmid^21^ and fused in-frame with an N-terminal six-histidine tag (His_6_) followed by a TEV (Tobacco Etch Virus) protease cleavage site. *Escherichia coli* BL21 (DE3) cells were transformed with RP1B-ARGLi and cultured at 37 °C until reaching optical density at 600 nm of ∼0.6. ARGLi expression was induced with 1 mM isopropyl-β-D-thiogalactopyranoside (IPTG) for 16 h at 30 °C. Cells were harvested by centrifugation and cell pellets were kept at −80 °C until protein purification.

### Purification of ARGLi

ARGLi-expressing cells were suspended in lysis buffer [50 mM Tris-HCl (pH 8.0), 500 mM NaCl, 5 mM imidazole, 0.1% triton-X 100, 250 μM phenylmethanesulfonyl (PMSF)] and lysed by sonication (15 cycles of 60 s sonication with 60 s intervals). The clarified cell lysate was filtered on 0.22 μm membrane and purified by nickel-affinity chromatography on a HisTrap HP column (GE Healthcare) pre-equilibrated in buffer A [50 mM Tris-HCl (pH 8.0), 500 mM NaCl, 5 mM imidazole, 10 mM β-mercaptoethanol]. On-column immobilised ARGLi was washed with 25 ml of buffer A containing 100 mM MnCl_2_ for enzyme activation. Elution was carried out with a linear imidazole gradient ranging from 5 to 500 mM. Fractions containing purified ARGLi, identified by enzyme activity and SDS-PAGE, were pooled, incubated with His_6_-TEV protease for removal of the N-terminal His_6_ tag, and dialysed against buffer [50 mM Tris-HCl (pH 7.4), 500 mM NaCl, 10 mM β-mercaptoethanol]. After complete cleavage, the His_6_ tag and His_6_-TEV protease were separated from ARGLi by a second nickel-affinity chromatography step, from which ARGLi flowed through the column. Finally, ARGLi was subjected to a final dialysis in buffer [50 mM CHES (pH 9.5), 5 mM DTT, 100 mM NaCl, 250 μM PMSF]. ARGLi purity was determined by SDS-PAGE and the enzyme was stored at −80 °C.

### ARGLi activity measurements

For the biochemical characterization and inhibition experiments, 0.2 μg/mL ARGLi was incubated with 50 mM L-arginine in 50 mM CHES buffer (pH 9.5) at 37 °C for 5 min. The amount of urea produced was determined by the UREA CE kit (Labtest), according to analytical procedures described by the manufacturer. Briefly, 10 μL of reaction mixture was incubated with 500 μL of urease solution (13 KU/L) diluted in buffer [20 mM sodium phosphate (pH 6.9), 62.4 mM sodium salicylate, 3 mM sodium nitroprusside] at 37 °C for 5 min. Subsequently, 500 μL of oxidising solution [140 mM sodium hydroxide, 6 mM sodium hypochlorite] was added and the indophenol blue formed was measured at 600 nm. The concentration of product was calculated using a standard curve of urea. Enzymatic assays were performed in triplicate.

### Determination of ARGLi optimal temperature and pH

To determine the influence of temperature on enzyme activity, 0.2 μg/mL ARGLi was incubated with 50 mM L-arginine in 50 mM CHES buffer (pH 9.5) for 5 min at different temperatures: 4 °C, 25 °C, 37 °C, 45 °C, 55 °C, 65 °C, 75 °C, and 85 °C. To determine the influence of pH, 0.2 μg/mL ARGLi was incubated with 50 mM L-arginine at 37 °C for 5 min under different buffer conditions:100 mM MOPS at pHs 7.0, 7.5, 8.0, and 100 mM CHES at pHs 8.6, 9.0, 9.5, and 10.0.

### Determination of ARGLi kinetic parameters

ARGLi (0.2 µg/mL) was incubated with L-arginine at concentrations ranging from 1 to 50 mM in 50 mM CHES buffer (pH 9.5) at 37 °C for 5 min. *V*_max_ and *K*_m_ values were determined by the analysis of the initial velocity versus substrate concentration curve using the Michaelis–Menten equation in Graph Prism 6.

### ARGLi inhibition by phenolic substances

**Figure 1.**
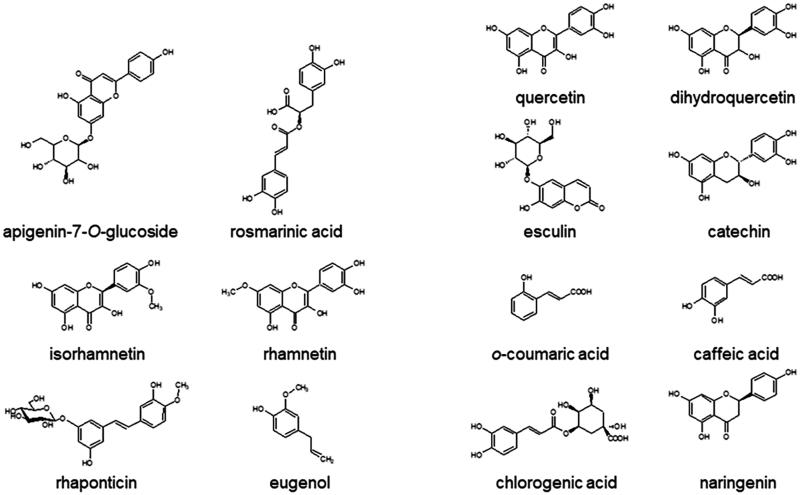
Chemical structures of natural phenolics tested against ARGLi activity.

A collection of fourteen phenolic substances containing flavonoids (apigenin-7-O-glucoside, catechin, dihydroquercetin, isorhamnetin, naringenin, quercetin, rhamnetin), stilbene (rhaponticin), phenylpropanoids (chlorogenic acid, eugenol, *o*-coumaric acid, rosmarinic acid), and coumarin (esculin) were investigated as ARGLi inhibitors (Figure 1). ARGLi (0.2 μg/mL) was incubated with 50 mM L-arginine (pH 9.5) at 37 °C for 5 min in the presence of 100 μM natural substances (diluted in DMSO). After reaction, the percentage of inhibition was calculated considering the enzyme activity of the control (absence of inhibitor) as 100%. Control reactions were performed in the presence of the same amount of DMSO (0.1%).

### Comparative modelling of ARGLi

*Leishmania infantum* arginase amino acid sequence was obtained from UniProtKB (access code A0A145YEM9)^22^. The template structure was obtained using standard options of BLASTP^23^ against the Protein Data Bank (PDB)^24^. Template selection considered the best results for identity, similarity, and gaps. Clustal-Omega (https://www.ebi.ac.uk/Tools/msa/clustalo/)^25^ was used for the alignment step and the three-dimensional model was built using MODELER 9 v19^26^. PROCHECK was used to evaluate the stereochemical quality of the final model^27^.

### Molecular docking

In order to establish and validate a protocol for molecular docking, the structure of NOR-N-OMEGA-HYDROXY-L-ARGININE (NNH) was re-docked into the crystallographic complex structure of *L. mexicana* arginase (PDB: 4IU1)^28^. Molecular docking calculations were performed in AutoDock 4.1. Lamarckian Genetic Algorithm was used and parameters such as initial population, number of energy assessments, mutation rate, crossover rate, elitism, and number of runs were modified. The active site was defined using AutoGrid. The grid size was set to 40 × 50 × 38 points with a grid spacing of 0.375 Å centred on the Nε of His139 residue in the crystal structure. The docking of each molecule consisted in a total of 100 runs that were carried out with an initial population of 150 individuals, a maximum of 240,000 energy evaluations, a maximum of 25,000 generations, a mutation rate of 0.02, an etilism of 1, and a crossover rate of 0.8. For the docking, ligand was considered flexible, while protein was treated as a rigid structure. Visual analysis of the generated complexes was performed using AutoDockTools 1.5.6^29^ and PyMOL (The PyMOL Molecular Graphics System, Version 1.4.1 Schrödinger, LLC.).

### *In silico* ADMET studies

*In silico* ADMET studies were performed using ADMET 8.5 (Simulations Plus, Inc., Lancaster, CA, USA), in which the Lipinski’s Rule of 5, toxicity evaluation, and ADMET risk were estimated for all substances exhibiting percentage of ARGLi inhibition higher than 50% at pH 7.4. As a control, the antileishmanial drug miltefosine was used.

### Parasites

*Leishmania (Leishmania) infantum* promastigotes, MHOM/BR/1974/PP75 strain, were obtained from the *Leishmania* Type Culture Collection (LTCC) of Oswaldo Cruz Institute/Fiocruz (Rio de Janeiro, RJ, Brazil). Parasites were maintained at 26 °C by weekly transfers in Schneider’s medium supplemented with 10% of inactivated FBS.

### Inhibition of *L. infantum* promastigotes growth

The parasite growth inhibition assays were performed on 96-well microplates, where the selected ARGLi inhibitors were previously diluted (1–200 μg/mL). Subsequently, promastigote forms of *L. infantum* collected in late logarithmic phase (96 h) were added to microplates at the final concentration of 10^6^ parasites/mL. Microplates were incubated at 26 °C for 96 h. After the incubation period, parasites viability was determined using 0.005% resazurin^30^. The 50% inhibitory concentration (IC_50_) was determined from the dose-response curves generated from the data.

### Cytotoxicity in RAW 264.7 macrophages

RAW 264.7 macrophages were cultured in polystyrene culture flasks containing DMEM medium supplemented with 10% FBS at 37 °C and 5% CO_2_ atmosphere. For cytotoxic evaluation, 48 h cultured cells were harvested, washed with culture medium, and distributed into 96-well culture plates at 10^5^ cells/well concentration. The cells were allowed to adhere for 2 h prior to the addition of increasing inhibitor concentrations (31–1000 µg/mL). Treated cell cultures were incubated for 48 h under the same experimental conditions. After this period, cell viability was determined by colorimetric assay using MTT solution (5 mg/mL) as an indicator. The 50% cytotoxic concentration (CC_50_) was calculated by analysing the dose-response curves generated from the data.

### Intracellular anti-amastigote activity and nitric oxide production by infected macrophages

The intracellular anti-amastigote activity was performed according to previously described procedures^31^ with some modifications. Initially, RAW 264.7 macrophages were distributed in 96-well plates and, after adherence (incubation for 2 h), cells were washed twice with phosphate buffer saline (PBS, pH 7.2). Then, the adherent cells were infected with promastigote forms of *L. infantum* at the stationary phase of growth in a proportion of 10 parasites/macrophage. After 4 h of interaction at 37 °C and 5% CO_2_ atmosphere, free parasites were washed away with PBS and infected macrophages were incubated for another 24 h to allow differentiation into amastigotes. After this time, infected cells were treated for 48 h with increasing concentrations of the selected inhibitor. Subsequently, the culture supernatant was collected for evaluation of nitric oxide production using the Griess reaction^32^. Cells were washed with PBS, then Schneider’s medium supplemented with 5% FBS was added and the plate was incubated at 26 °C for 72 h. The parasite survival was estimated by viability of differentiated promastigote forms recovered from the cultures using MTT (5 mg/mL). The results were expressed as the percentage of viability in relation to that obtained for the control (100%). The IC_50_ for amastigote forms was calculated from the dose-response curves generated from the data.

### Selectivity index

The selectivity index (SI) for promastigotes and intracellular amastigotes of *L. infantum* was calculated by the ratio between the CC_50_ obtained for the host cell and the parasite IC_50_. Inhibitors that showed SI ≥10 were considered low cytotoxic^33^.

## Results and discussion

### Optimal parameters and kinetic properties of ARGLi

**Figure 2.**
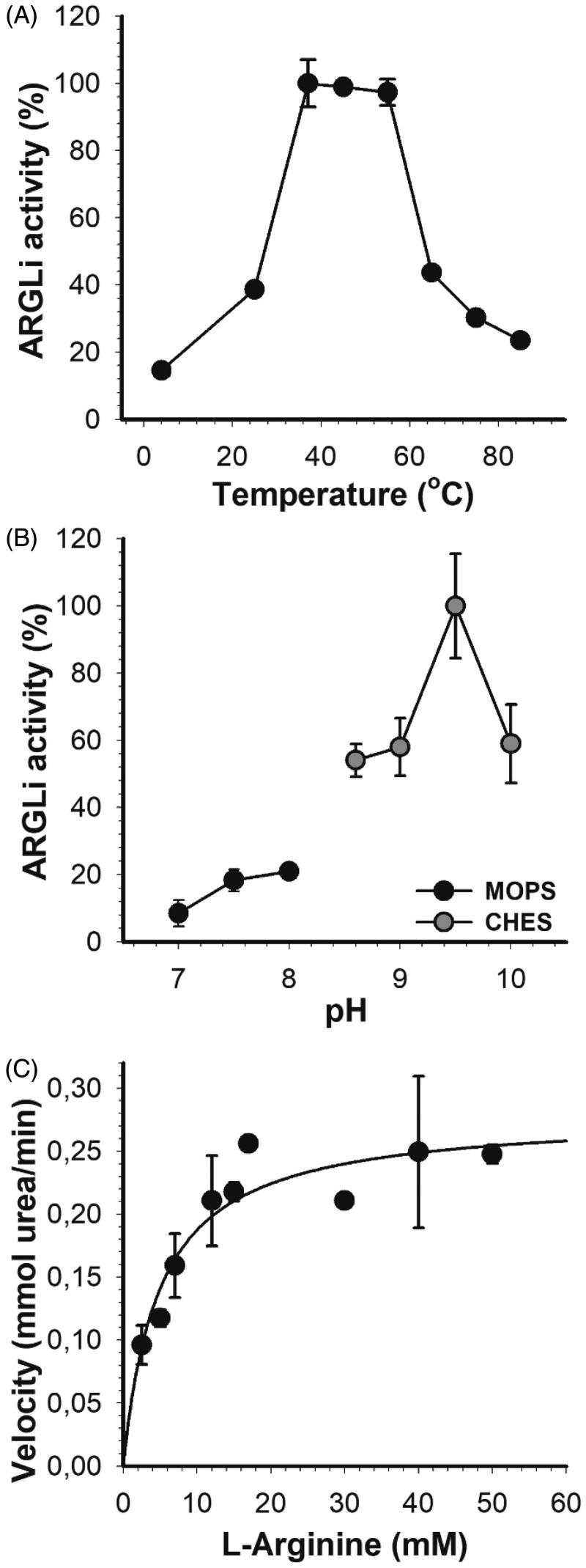
Biochemical characterization of ARGLi. (A) Effect of temperature on ARGLi activity. ARGLi-catalyzed urea production was measured over different temperatures ranging from 5 °C to 85 °C. (B) Effect of pH on ARGLi activity. ARGLi-catalysed urea production was measured over a pH range of 7.0–10.0. MOPS was used as a buffer for pHs 7.0–8.0, while CHES was used for pHs 8.6–10.0. (C) Michaelis–Menten kinetics of ARGLi. Initial velocity was plotted against the concentration of L-arginine. Kinetic parameters (*V*_max_ of 0.28 ± 0.016 mM/min; *K*_m_ of 5.1 ± 1.1 mM) were determined from the non-linear regression of the Michaelis–Menten curve. Data represent the mean ± SE of three independent measurements.

In order to investigate the inhibitory profile of ARGLi by natural substances, we first characterised its optimal parameters and kinetic properties. ARGLi showed the highest activity between 37 °C and 55 °C, with an activity drop at temperatures above 60 °C (Figure 2(A)). Similar temperature conditions have been described for other isoforms of the enzyme^34^. We then selected the temperature of 37 °C for all subsequent enzyme activity measurements.

ARGLi displayed a stringent pH dependency centred at pH 9.5 (Figure 2(B)). A difference in ±0.5 pH units caused a sharp decrease in about 50% of enzyme activity. Similar results were found for arginase from other *Leishmania* species. Recombinant arginase from *L. mexicana* showed a broad pH optimum between 8.5 and 9.5^11^. Recombinant *L. amazonensis* arginase exhibited the highest activity at pH 9.6. A decrease in reaction pH to 7.0 led to an increase in enzyme *K*_m_ accompanied by a decrease in *V*_max_^12^. Recombinant arginase from human liver and arginase isolated from erythrocytes also showed basic pH optima with values ranging from 9.7 to 11^34^. Thus, our results agree well with those previously reported, in which basic pH 9.5 or close is ideal for performing arginase activity measurements.

ARGLi exhibited classical Michaelis–Menten kinetics, a hyperbolic dependency of the reaction rate as a function of L-arginine concentration. Non-linear regression of the Michaelis–Menten plot enabled the determination of kinetic parameters, such as *V*_max_ (0.28 ± 0.016 mM/min) and *K*_m_ (5.1 ± 1.1 mM) (Figure 2(C)). ARGLi *K*_m_ values are more similar to those described for human arginase I (*K*_m_=7.6 mM)^35^ than for native and recombinant *L. amazonensis* arginase (*K*_m_=23.9 ± 0.96 and 21.5 ± 0.9, respectively)^12^, recombinant *L. mexicana* arginase (*K*_m_=25 ± 4 mM)^11^, and recombinant human arginase (*K*_m_=13 ± 2 mM)^11^. Our results suggest that ARGLi shows a stronger affinity for L-arginine than arginase from human and tegumentary *Leishmania* species. From the *K*_m_ and *V*_max_ values, we calculated a *K*_cat_ of 2.55 × 10^3^ s^−1^ and a specificity constant (*K*_cat_/*K*_m_) of 5 × 10^8^ M^−1^s^−1^. The high *K*_cat_ and *K*_cat_/*K*_m_ values suggest that ARGLi displays excellent catalytic efficiency, higher than that described for *L. mexicana* arginase (*K*_cat_=1.7 s^−1^)^11^.

### ARGLi inhibition by natural phenolic substances

**Figure 3.**
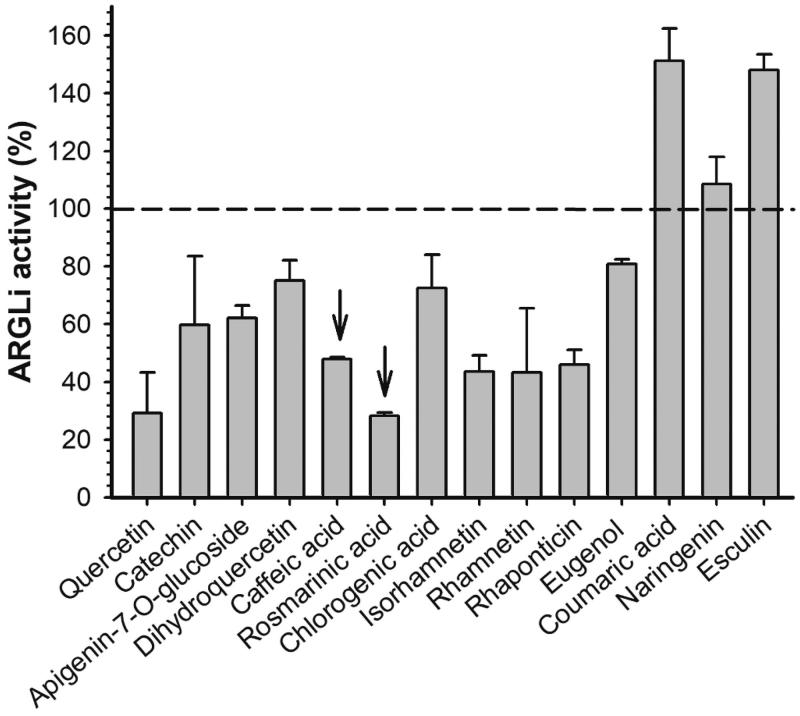
Effect of phenolic substances of natural origin on ARGLi activity. ARGLi-catalysed urea production was measured in the absence (control) and presence of fourteen natural phenolics at 100 μM concentration. Data are shown as percentage of ARGLi activity in relation to the control and represent the mean ± SE of three independent measurements. The dashed line highlights 100% ARGLi activity (control). Arrows indicate rosmarinic acid (71.48 ± 0.85%) and caffeic acid (56.98 ± 5.51%) as potent ARGLi inhibitors.

The previously reported *L. amazonensis* arginase inhibitors of natural origin concentrate in the group of phenolic substances, mainly flavonoids^12–16^. Therefore, quercetin and catechin were used as positive controls of ARGLi inhibition. These substances were previously described as *L. amazonensis* arginase inhibitors and are widely known as leishmanicidal agents^12^^,^^14^. Quercetin and catechin (100 µM), inhibited ARGLi activity by 67.05 ± 10.36% and 49.02 ± 14.92%, respectively. Among the phenolic substances tested against ARGLi, we highlight the phenylpropanoids rosmarinic acid and caffeic acid (100 µM), which showed percentages of inhibition of 71.48 ± 0.85% and 56.98 ± 5.51%, respectively (Figure 3).

Interestingly, many of the phenolic substances that exhibited the greatest inhibitory activity against ARGLi (quercetin, catechin, caffeic acid, rosmarinic acid, chlorogenic acid, and rhamnetin) share a common structural characteristic; they all contain a catechol group. The importance of the catechol group for efficient arginase inhibition has been described for other *Leishmania* species. Chlorogenic acid, (+)-catechin, (−)-epicatechin, and isoquercitrin, isolated from the ethyl acetate fraction of *Cecropia pachystachya* leaf extract, inhibited more than 50% of *L. amazonensis* arginase activity at a final concentration of 20 μM^13^. In addition to these phenolics, orientin, isoorientin, fisetin, and luteolin showed inhibitory activity against *L. amazonensis* arginase with IC_50_ values of 16 ± 2.0, 9.0 ± 1.0, 1.4 ± 0.3, and 9.0 ± 1.0 μM, respectively^15^. The high activity displayed by these substances was attributed to the presence of the catechol group, since other phenolics that do not contain a catechol group in their structures (apigenin, vitexin, and isovitexin) showed little effect. Comparative analysis of the structure and inhibitory activity of quercetin (IC_50_=4.3 μM) with galangin (IC_50_=100 μM) and kaempferol (IC_50_=50 μM) revealed the importance of the phenolic group hydroxylation for increased arginase inhibition^15^. In addition, by investigating the interaction of (+)-catechin and (−)-epicatechin with *L. amazonensis* arginase *in silico*, dos Reis et al. showed that the catechol group makes hydrogen bonds with amino acids from the enzyme active site^14^. These phenolics also showed interaction with the arginase cofactor, acting as manganese chelators^12^. The relationship between the presence of the catechol group and the inhibitory activity was also observed against *L. donovani*, in which quercetin (IC_50_=1 μg/mL) was three times more active than kaempferol (IC_50_=2.9 μg/mL)^36^.

Caffeic acid and quercetin (100 μM) have been shown to inhibit bovine hepatic arginase with percentages of inhibition and IC_50_ values of 61.3 ± 4.1% and 86.7 μM for caffeic acid, and 64.4 ± 2.5% and 31.2 μM for quercetin^37^. Concomitant inhibition of *Leishmania* and mammalian arginase may represent a potential therapeutic strategy, since inhibition of host arginase may increase NO production^38^^,^^39^.

### Structural investigation of rosmarinic acid interaction with ARGLi

An experimentally-derived, atomic-resolution structure of *L. infantum* arginase has not been reported so far. Thus, we used comparative modelling to build a three-dimensional structural model of ARGLi, enabling us to investigate its interaction with inhibitors. The accuracy of protein structure models depends on the identity between the template and target sequences. We used the crystal structure of *L. mexicana* arginase in complex with the NNH inhibitor (PDB: 4IU1)^28^, which showed 96% of identity with the target sequence, as a template. The sequence alignment obtained from Clustal-Omega was provided as input in MODELLER to generate the three-dimensional model of ARGLi. The final model displayed 91.1% residues in the most favourable regions, 8.2% in allowed regions, 0.4% in generously allowed regions, and 0.4% in disallowed regions of the Ramachandran plot (Supplemental Figure S1). As Lys9 was the only residue in the disallowed region of the Ramachandran plot and it is located far from the enzyme active site, the model was considered sufficient for further studies.

In order to gain insights into the mechanism of ARGLi inhibition, we investigated its interaction with rosmarinic acid by molecular docking. Initially, the docking accuracy was evaluated by redocking the NNH inhibitor into the crystal structure of *L. mexicana* arginase (PDB: 4IU1)^28^. The *in silico* analysis revealed a conformation similar to the crystal structure with a root mean square deviation (RMSD) between the top docking pose and the original crystallographic geometry of 0.4 Å (Supplemental Figure S2). This result supported the hypothesis that the docking protocol was able to reproduce the experimental binding mode. Thus, the same docking procedure was employed for rosmarinic acid.

**Figure 4.**
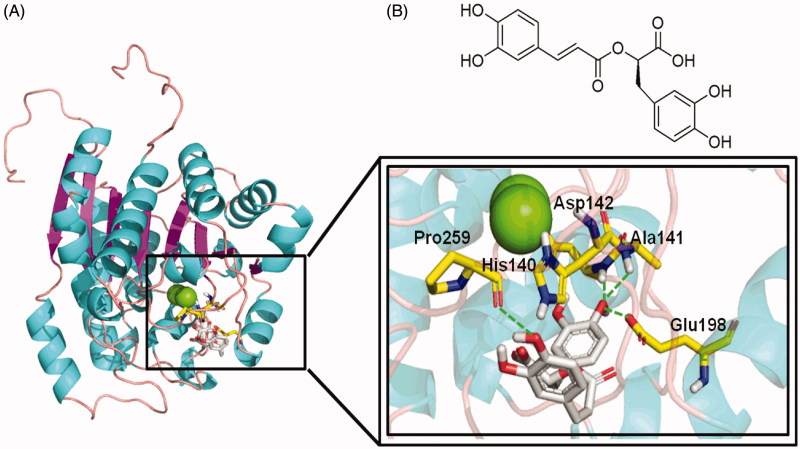
Rosmarinic acid mode of binding to ARGLi active site. (A) Close-up view of rosmarinic acid docked into the active site of ARGLi. ARGLi three-dimensional model is shown in cartoon representation. α-Helices are coloured cyan, β-sheets are coloured magenta, and loops are coloured light pink. The two manganese ions inside ARGLi active site are displayed as green spheres. Residues His140, Ala141, Asp142, Glu198, and Pro259, which directly interact with rosmarinic acid (gray), are displayed in yellow and marked. Hydrogen bonds are represented as green dashed lines. (B) Chemical structure of rosmarinic acid.

The ARGLi-rosmarinic acid complex showed an estimated binding affinity energy of −45 kcal/mol. Four hydrogen bonds were observed between the inhibitor and ARGLi. The hydroxyl oxygen atom at para position of the ligand ring B (catechol group) with the main chain nitrogen atoms of residues Ala141 (distance O···N 2.2 Å) and Asp142 (distance O···N 2.4 Å), and the side chain oxygen atom of residue Glu198 (distance O···O 2.0 Å) and the main chain oxygen atom of residue Pro259 (distance O···O 2.4 Å) with the hydroxyl oxygen atom at para position of the ligand ring A. In addition, cation-π interactions between the aromatic ring of the substance and residue His140 (distance 2.4 Å) were identified (Figure 4). It is worth noting that His140, Ala141, and Asp142 are conserved among arginases and are key residues for ligand interaction with *L. mexicana* arginase (His139, Ala140, and Asp141, respectively)^28^^,^^40^.

Finally, ADMET properties were estimated for caffeic acid, isorhamnetin, rhamnetin, rhaponticin, and rosmarinic acid, since these substances inhibited more than 50% of ARGLi activity. Our results suggested that these substances exhibit good oral bioavailability, based on the Lipinski rule of 5^41^. In the toxicity evaluation, these substances displayed low risk of cardiotoxicity. In contrast, except for rosmarinic acid, they showed a high risk of hepatotoxicity and mutagenicity. In addition, pharmacokinetic and toxicological parameters were compiled in ADMET Risk. The substances showed a value in the range of 2.01–5.68. ADMET risk provides a range between 0 and 24. These values indicate the number of potential ADMET risk factors that a compound might possess. Thus, we may infer that all substances exhibit low probability of having ADMET problems.

### *In vivo* activity of ARGLi inhibitors

Substances that inhibited more than 50% of ARGLi activity were selected for the *in vivo* assays against *L. infantum* parasites. Except for rhaponticin, which did not inhibit promastigote growth at the highest concentration tested (Table 1), all other ARGLi inhibitors displayed antileishmanial activity. The phenylpropanoids rosmarinic acid and caffeic acid displayed the greatest effects against *L. infantum* with IC_50_ values of 57.3 ± 2.65 μM (20.64 ± 0.96 µg/mL) and 60.8 ± 11 μM (10.97 ± 2.01 µg/mL) for promastigotes, and 7.9 ± 1.7 µM (2.86 ± 0.62 µg/mL) and 21.9 ± 5.0 µM (3.95 ± 0.91 µg/mL) for intracellular amastigotes, respectively (Table 1). Montrieux et al. previously described the leishmanicidal activity of rosmarinic acid and caffeic acid against the dermotropic species *L. amazonensis in vitro* and *in vivo*. These phenolic acids displayed IC_50_ values of 0.2 ± 0.1 and 0.9 ± 0.2 µg/mL, respectively, against promastigote forms^42^. Caffeic acid showed an IC_50_ of 2.9 ± 0.3 µg/mL for intracellular amastigotes of *L. amazonensis* and a CC_50_ of 32.5 ± 2.0 µg/mL for BALB/c mice peritoneal macrophages. Comparatively, rosmarinic acid was more active and showed greater selectivity for *L. amazonensis* than caffeic acid, exhibiting an IC_50_ of 1.7 ± 0.4 µg/mL for intracellular amastigotes and a CC_50_ of 33.5 ± 1.4 µg/mL^42^. In addition, these phenolic acids proved to be more effective in reducing lesion size and parasite burden of *L. amazonensis*-infected BALB/c mice than the reference drug Glucantime^®^. Moreover, treatment with these substances did not lead to mice death or significant loss of body weight^42^. Previous reports have demonstrated the anti-*L. infantum* activity of plant extracts enriched in rosmarinic and caffeic acids^43–45^. Here, we demonstrate the inhibitory effect of these phenylpropanoids alone against this viscerotropic species.

**Table 1.** Activity of ARGLi inhibitors against RAW 264.7 macrophages (CC_50_±SE) as well as *L. infantum* promastigotes and intracellular amastigotes (IC_50_±SE).

Inhibitor	RAW 264.7		Promastigotes		Intracellular amastigotes		SI	
	CC_50_±SE (µg/mL)	CC_50_±SE (µM)	IC_50_±SE (µg/mL)	IC_50_±SE (µM)	IC_50_±SE (µg/mL)	IC_50_±SE (µM)	PRO	AMA
Catechin	256.98 ± 0.64	885 ± 2.5	114.8 ± 14.65	395 ± 50	83.28 ± 10.6	286.9 ± 36.5	2.23	3.08
Caffeic acid	220 ± 4.99	1221 ± 28	10.97 ± 2.01	60.8 ± 11	3.95 ± 0.91	21.9 ± 5.0	20.05	55.69
Rosmarinic acid	176.74 ± 14.9	491 ± 41.5	20.64 ± 0.96	57.3 ± 2.65	2.86 ± 0.62	7.9 ± 1.7	8.56	61.79
Isorhamnetin	>1000	>3000	258.7 ± 9.50	818 ± 30	n.d.	n.d.	n.d.	n.d.
Rhamnetin	>1000	>3000	263.2 ± 3.87	832 ± 6.9	n.d.	n.d.	n.d.	n.d.
Rhaponticin	825.27 ± 15.68	1963 ± 38	>400	>1000	n.d.	n.d.	n.d.	n.d.
Fungizone	11.07 ± 0.17	11.97 ± 0.2	0.04 ± 0.006	0.05 ± 0.006	0.18 ± 0.025	0.191 ± 0.02	251.6	62.54

n.d: non-determined; SI: selectivity index (CC_50_/IC_50_); PRO: promastigotes; AMA: intracellular amastigotes.

Rhamnetin and isorhamnetin, although inhibiting ∼56% of arginase activity, showed low activity against promastigote forms of *L. infantum* with IC_50_ of 832 ± 6.9 μM (258.7 ± 9.5 μg/mL) and 818 ± 30 μM (263.2 ± 3.9 μg/mL), respectively. Despite the low activity, these substances did not exhibit toxicity for RAW 264.7 macrophages at the highest concentration tested (1000 μg/mL) and thus they are expected to be highly selective. Tasdemir et al. described better results for rhamnetin and isorhamnetin using axenic amastigote forms of *L. donovani*^36^. Both isorhamnetin (IC_50_=3.8 μg/mL) and rhamnetin (IC_50_=4.6 μg/mL) showed greater selectivity for the parasite than for myoblast lineage cells (L9) with SI of 10.7 and >20, respectively.

Catechin displayed a CC_50_ of 885 ± 2.5 µM (256.9 ± 0.6 μg/mL) and IC_50_ of 395 ± 50 µM (114.7 ± 14.6 μg/mL) for *L. infantum* promastigotes and 286.9 ± 36.5 µM (83.2 ± 10.6 μg/mL) for intracellular amastigotes, leading to SI values of 2.23 and 3.08, respectively. Extraction of *Rhus punjabensis* leaves in methanol/chloroform originated a catechin-rich extract that showed an IC_50_ of 77.75 ± 1.23 μg/mL for promastigotes forms of *L. tropica*^46^. In addition, catechin isolated from the ethanolic extract of *Stryphnodendron obovatum* stem bark showed an IC_50_ of 43.2 ± 2.1 μg/mL for *L. amazonensis* promastigotes and a SI of 2.7^47^. These results suggest that catechin displays higher activity against *Leishmania* spp. responsible for the tegumentary forms of the disease.

Despite presenting the lowest IC_50_ for *L. infantum* amastigotes (0.191 ± 0.02 μM), the reference drug Fungizone^®^ showed a SI of 62.54, which is quite similar to those found for rosmarinic acid (61.79) and caffeic acid (55.69). Therefore, the pronounced antileishmanial activity of these substances in combination with their greater selectivity for the parasites evidence their potential use as promising candidates for less toxic and more effective drugs in VL treatment.

**Figure 5.**
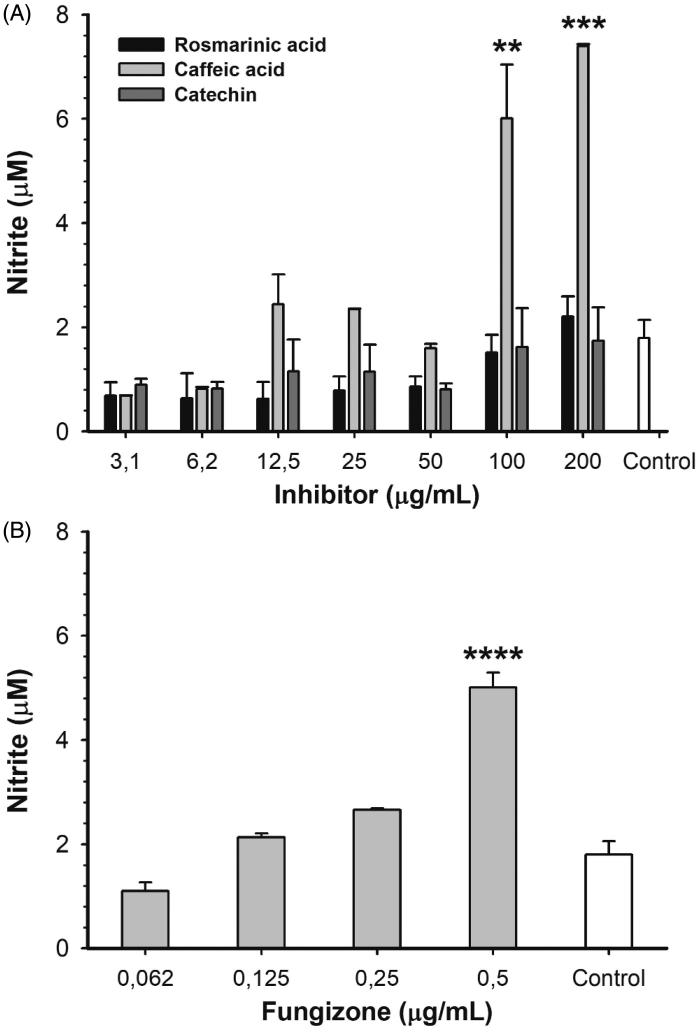
Nitric oxide production by *L. infantum*-infected RAW 264.7 macrophages treated with different concentrations of ARGLi inhibitors. (A) Treatment with 3.1–200 μg/mL of rosmarinic acid (black), caffeic acid (light gray), and catechin (dark gray). (B) Treatment with 0.062–0.5 μg/mL of the reference drug fungizone. Control (white) represents the production of nitric oxide by infected and untreated macrophages. Nitrite concentration was measured by the Griess reaction and data represent mean ± SE of two independent experiments. Asterisks indicate treatments that were significantly different compared to the control, in which ****p < .0001,***p < .001, and **p < .01.

In order to determine if ARGLi inhibitors were capable of modulating the host cell response, RAW 264.7 macrophages were infected with promastigote forms of *L. infantum* and treated with different concentrations of inhibitors for 48 h. After treatment, NO production was measured^32^. Among the inhibitors tested (catechin, rosmarinic acid, and caffeic acid), only caffeic acid significantly increased NO production. Treatment with 100 and 200 µg/mL caffeic acid induced an increase in nitrite levels of 234.6% (*p* > .01) and 311.5% (*p* > .001) in comparison to the control, respectively (Figure 5(A)). This increase is similar to that observed for the reference drug Fungizone^®^ (Figure 5(B)). The effect of caffeic acid on NO production may be a result of macrophage arginase inhibition and consequent oxidation of accumulated L-arginine by inducible NO synthase (iNOS). Caffeic acid has been shown to positively regulate iNOS expression and activity in a TNFα-dependent manner in *L. major*-infected BALB/c mice^48^. Remarkably, despite their anti-amastigote activity, catechin and rosmarinic acid did not affect NO production. These results suggest that catechin and rosmarinic acid trigger a NO-independent antileishmanial mechanism, which may include ARGLi inhibition *in vivo*.

Our results reinforce the importance of the catechol group for ARGLi inhibition. Catechol groups qualify as pan assay interference compounds (PAINS), due to their redox activity and reactivity against proteins^49^. Even though discriminating between false positives and true hits is a complex and difficult task, evidence of specific interactions between substance and target as well as a description of the mechanism of action validate compounds inhibitory activity. Previous results have shown that the catechol-containing norathyriol is a site-specific inhibitor of mitogen-activated protein kinase 1 (ERK2), further supporting its potential activity^50^. Our *in silico* docking results revealed that rosmarinic acid makes a number of direct contacts with ARGLi, suggesting specific interactions. In addition, we conducted an in-depth *in vivo* inhibition study that showed that caffeic acid acts by increasing NO production by infected macrophages, even suggesting inhibition of host arginase.

## Conclusion

We report on the purification, biochemical characterization, and inhibition of recombinant arginase from *L. infantum* (ARGLi). The enzyme showed a strong affinity for L-arginine and excellent catalytic efficiency. Among the phenolic substances tested, the phenylpropanoids rosmarinic acid and caffeic acid displayed potent inhibitory activity. Rosmarinic acid and caffeic acid were effective against *L. infantum* promastigote and intracellular amastigote forms and exhibited high selectivity. Moreover, caffeic acid led to an increase in NO production by infected macrophages. The results presented here further support arginase inhibition by naturally occurring phenolics as a promising strategy for VL treatment. It should be also noted that these natural products show effective inhibition of other enzymes such as for example the carbonic anhydrases^51–55^. As *Leishmania* spp. also encode for such an enzyme^56–60^, our findings may provide the rationale for using such multi-targeted derivatives for the management of this protozoan infection.

## Supplementary Material

Supplemental Material

## References

[1] Torres-Guerrero E, Quintanilla-Cedillo MR, Ruiz-Esmenjaud J, et al. Leishmaniasis: a review. F1000Res 2017;6:750. 28649370 10.12688/f1000research.11120.1 PMC5464238

[2] Alvar J, Vélez ID, Bern C, et al. Leishmaniasis worldwide and global estimates of its incidence. PLoS One 2012;7:e35671. 22693548 10.1371/journal.pone.0035671 PMC3365071

[3] Das A, Karthick M, Dwivedi S, et al. Epidemiologic correlates of mortality among symptomatic visceral leishmaniasis cases: findings from situation assessment in high endemic foci in India. PLoS Neglect Trop Dis 2016;10:e0005150. 10.1371/journal.pntd.0005150 PMC5117587 27870870

[4] World Health Organization (WHO). Global Health Observatory (GHO) data. Leishmaniasis: Situation and trends. Available from https://www.who.int/gho/neglected_diseases/leishmaniasis/en/. [last accessed 23 Feb 2019].

[5] Uliana SRB, Trinconi CT, Coelho AC Chemotherapy of leishmaniasis: present challenges. Parasitology 2018;145:464–80. 28103966 10.1017/S0031182016002523

[6] Christianson DW Arginase: structure, mechanism, and physiological role in male and female sexual arousal. Acc Chem Res 2005;38:191–201. 15766238 10.1021/ar040183k

[7] Munder M Arginase: an emerging key player in the mammalian immune system. Br J Pharmacol 2009;158:638–51. 19764983 10.1111/j.1476-5381.2009.00291.x PMC2765586

[8] Roberts SC, Tancer MJ, Polinsky MR, et al. Arginase plays a pivotal role in polyamine precursor metabolism in *Leishmania*. Characterization of gene deletion mutants. J Biol Chem 2004;279:23668–78. 15023992 10.1074/jbc.M402042200

[9] Aoki JI, Muxel SM, Zampieri RA, et al. RNA-seq transcriptional profiling of *Leishmania amazonensis* reveals an arginase-dependent gene expression regulation. PLoS Negl Trop Dis 2017;11:e0006026. 29077741 10.1371/journal.pntd.0006026 PMC5678721

[10] Colotti G, Ilari A Polyamine metabolism in *Leishmania*: from arginine to trypanothione. Amino Acids 2011;40:269–85. 20512387 10.1007/s00726-010-0630-3

[11] Riley E, Roberts SC, Ullman B Inhibition profile of *Leishmania mexicana* arginase reveals differences with human arginase I. Int J Parasitol 2011;41:545–52. 21232540 10.1016/j.ijpara.2010.12.006 PMC3062745

[12] da Silva ER, Maquiaveli CC, Magalhães PP The leishmanicidal flavonols quercetin and quercitrin target *Leishmania* (*Leishmania*) *amazonensis* arginase. Exp Parasitol 2012;130:183–8. 22327179 10.1016/j.exppara.2012.01.015

[13] Cruz EM, da Silva ER, Maquiaveli CC, et al. Leishmanicidal activity of *Cecropia pachystachya* flavonoids: arginase inhibition and altered mitochondrial DNA arrangement. Phytochemistry 2013;89:71–7. 23453911 10.1016/j.phytochem.2013.01.014

[14] dos Reis MB, Manjolin LC, Maquiaveli CC, et al. Inhibition of *Leishmania* (*Leishmania*) *amazonensis* and rat arginases by green tea EGCG, (+)-catechin and (-)-epicatechin: a comparative structural analysis of enzyme-inhibitor interactions. PLoS One 2013;8:e78387. 24260115 10.1371/journal.pone.0078387 PMC3832641

[15] Manjolin LC, dos Reis MB, Maquiaveli CC, et al. Dietary flavonoids fisetin, luteolin and their derived compounds inhibit arginase, a central enzyme in *Leishmania* (*Leishmania*) *amazonensis* infection. Food Chem 2013;141:2253–62. 23870955 10.1016/j.foodchem.2013.05.025

[16] de Sousa LR, Ramalho SD, Burger MC, et al. Isolation of arginase inhibitors from the bioactivity-guided fractionation of *Byrsonima coccolobifolia* leaves and stems. J Nat Prod 2014;77:392–6. 24521209 10.1021/np400717m

[17] da Silva ER, Boechat N, Pinheiro LC, et al. Novel selective inhibitor of *Leishmania* (*Leishmania*) *amazonensis* arginase. Chem Biol Drug Des 2015;86:969–78. 25845502 10.1111/cbdd.12566

[18] Maquiaveli CC, Lucon-Júnior JF, Brogi S, et al. Verbascoside inhibits promastigote growth and arginase activity of *Leishmania amazonensis*. J Nat Prod 2016;79:1459–63. 27096224 10.1021/acs.jnatprod.5b00875

[19] Lacerda RBM, Freitas TR, Martins MM, et al. Isolation, leishmanicidal evaluation and molecular docking simulations of piperidine alkaloids from *Senna spectabilis*. Bioorg Med Chem 2018;26:5816–23. 30413343 10.1016/j.bmc.2018.10.032

[20] Adinehbeigi K, Razi Jalali MH, Shahriari A, et al. In vitro antileishmanial activity of fisetin flavonoid via inhibition of glutathione biosynthesis and arginase activity in *Leishmania infantum*. Pathog Glob Health 2017;111:176–85. 28385129 10.1080/20477724.2017.1312777 PMC5498762

[21] Peti W, Page R Strategies to maximize heterologous protein expression in *Escherichia coli* with minimal cost. Protein Expr Purif 2007;51:1–10. 16904906 10.1016/j.pep.2006.06.024

[22] UniProt Consortium Reorganizing the protein space at the Universal Protein Resource (UniProt). Nucleic Acids Res 2012;40:D71–75. 22102590 10.1093/nar/gkr981 PMC3245120

[23] Altschul SF, Madden TL, Schäffer AA, et al. Gapped BLAST and PSI-BLAST: a new generation of protein database search programs. Nucleic Acids Res 1997;25:3389–402. 9254694 10.1093/nar/25.17.3389 PMC146917

[24] Berman HM, Westbrook J, Feng Z, et al. The protein data bank. Nucleic Acids Res 2000;28:235–42. 10592235 10.1093/nar/28.1.235 PMC102472

[25] Sievers F, Wilm A, Dineen D, et al. Fast, scalable generation of high-quality protein multiple sequence alignments using Clustal Omega. Mol Syst Biol 2014;7:539. 10.1038/msb.2011.75 PMC3261699 21988835

[26] Sali A, Blundell TL Comparative protein modelling by satisfaction of spatial restraints. J Mol Biol 1993;234:779–815. 8254673 10.1006/jmbi.1993.1626

[27] Laskowski RA, MacArthur MW, Moss DS, et al. PROCHECK: a program to check the stereochemical quality of protein structures. J Appl Cryst 1993;26:283–91.

[28] D'Antonio EL, Ullman B, Roberts SC, et al. Crystal structure of arginase from *Leishmania mexicana* and implications for the inhibition of polyamine biosynthesis in parasitic infections. Arch Biochem Biophys 2013;535:163–76. 23583962 10.1016/j.abb.2013.03.015 PMC3683356

[29] Morris GM, Huey R, Lindstrom W, et al. Autodock4 and AutoDockTools4: automated docking with selective receptor flexibility. J Comput Chem 2009;30:2785–91. 19399780 10.1002/jcc.21256 PMC2760638

[30] Rolón M, Vega C, Escario JA, et al. Development of resazurin microtiter assay for drug sensibility testing of *Trypanosoma cruzi* epimastigotes. Parasitol Res 2006;99:103–7. 16506080 10.1007/s00436-006-0126-y

[31] Passero LF, Bonfim-Melo A, Corbett CE, et al. Anti-leishmanial effects of purified compounds from aerial parts of *Baccharis uncinella* C. DC. (Asteraceae). Parasitol Res 2011;108:529–36. 20886232 10.1007/s00436-010-2091-8

[32] Misko TP1, Schilling RJ, Salvemini D, et al. A fluorometric assay for the measurement of nitrite in biological samples. Anal Biochem 1993;214:11–6. 7504409 10.1006/abio.1993.1449

[33] Katsuno K, Burrows JN, Duncan K, et al. Hit and lead criteria in drug discovery for infectious diseases of the developing world. Nat Rev Drug Discov 2015;14:751–8. 26435527 10.1038/nrd4683

[34] Ikemoto M, Tabata M, Miyake T, et al. Expression of human liver arginase in *Escherichia coli*. Purification and properties of the product. Biochem J 1990;270:697–703. 2241902 10.1042/bj2700697 PMC1131788

[35] Di Costanzo L, Moulin M, Haertlein M, et al. Expression, purification, assay, and crystal structure of perdeuterated human arginase I. Arch Biochem Biophys 2007;465:82–9. 17562323 10.1016/j.abb.2007.04.036 PMC2018606

[36] Tasdemir D, Kaiser M, Brun R, et al. Antitrypanosomal and antileishmanial activities of flavonoids and their analogues: in vitro, in vivo, structure-activity relationship, and quantitative structure-activity relationship studies. Antimicrob Agents Chemother 2006;50:1352–64. 16569852 10.1128/AAC.50.4.1352-1364.2006 PMC1426963

[37] Bordage S, Pham TN, Zedet A, et al. Investigation of mammal arginase inhibitory properties of natural ubiquitous polyphenols by using an optimized colorimetric microplate assay. Planta Med 2017;83:647–53. 27776374 10.1055/s-0042-118711

[38] Iniesta V, Gómez-Nieto LC, Corraliza I The inhibition of arginase by N(omega)-hydroxy-l-arginine controls the growth of *Leishmania* inside macrophages. J Exp Med 2001;193:777–84. 11257143 10.1084/jem.193.6.777 PMC2193414

[39] Kropf P, Fuentes JM, Fähnrich E, et al. Arginase and polyamine synthesis are key factors in the regulation of experimental leishmaniasis in vivo. Faseb J 2005;19:1000–2. 15811879 10.1096/fj.04-3416fje

[40] Ash DE Structure and function of arginases. J Nutr 2004;134:2760S–4S. 15465781 10.1093/jn/134.10.2760S

[41] Lipinski CA Lead- and drug-like compounds: the rule-of-five revolution. Drug Discov Today Technol 2004;1:337–41. 24981612 10.1016/j.ddtec.2004.11.007

[42] Montrieux E, Perera WH, García M, et al. In vitro and in vivo activity of major constituents from *Pluchea carolinensis* against *Leishmania amazonensis*. Parasitol Res 2014;113:2925–32. 24906989 10.1007/s00436-014-3954-1

[43] Brito SM, Coutinho HD, Talvani A, et al. Analysis of bioactivities and chemical composition of *Ziziphus joazeiro* Mart. using HPLC-DAD. Food Chem 2015;186:185–91. 25976809 10.1016/j.foodchem.2014.10.031

[44] Cunha F, Tintino SR, Figueredo F, et al. HPLC-DAD phenolic profile, cytotoxic and anti-kinetoplastidae activity of *Melissa officinalis*. Pharm Biol 2016;54:1664–70. 26864563 10.3109/13880209.2015.1120320

[45] Calixto Júnior JT, de Morais SM, Gomez CV, et al. Phenolic composition and antiparasitic activity of plants from the Brazilian Northeast "Cerrado". Saudi J Biol Sci 2016;23:434–40. 27081371 10.1016/j.sjbs.2015.10.009 PMC4818332

[46] Tabassum S, Ahmed M, Mirza B, et al. Appraisal of phytochemical and in vitro biological attributes of an unexplored folklore: Rhus Punjabensis Stewart. BMC Complement Altern Med 2017;17:146. 28274230 10.1186/s12906-017-1659-6 PMC5343295

[47] Ribeiro TG, Nascimento AM, Henriques BO, et al. Antileishmanial activity of standardized fractions of *Stryphnodendron obovatum* (Barbatimão) extract and constituent compounds. J Ethnopharmacol 2015;165:238–42. 25732835 10.1016/j.jep.2015.02.047

[48] Belkhelfa-Slimani R, Djerdjouri B Caffeic acid and quercetin exert caspases-independent apoptotic effects on *Leishmania* major promastigotes, and reactivate the death of infected phagocytes derived from BALB/c mice. Asian Pac J Trop Biomed 2017;7:321–31.

[49] Baell JB, Holloway GA New substructure filters for removal of pan assay interference compounds (PAINS) from screening libraries and for their exclusion in bioassays. J Med Chem 2010;53:2719–40. 20131845 10.1021/jm901137j

[50] Li J, Malakhova M, Mottamal M, et al. Norathyriol suppresses solar UV-induced skin cancer by targeting ERKs. Cancer Res 2012;72:260–70. 22084399 10.1158/0008-5472.CAN-11-2596 PMC3251698

[51] (a) Supuran CT Structure and function of carbonic anhydrases. Biochem J 2016;473:2023–32. 27407171 10.1042/BCJ20160115

[52] (a) Capasso C, Supuran CT An overview of the alpha-, beta-and gamma-carbonic anhydrases from bacteria: can bacterial carbonic anhydrases shed new light on evolution of bacteria? J Enzyme Inhib Med Chem 2015;30:325–32. 24766661 10.3109/14756366.2014.910202

[53] (a) Briganti F, Pierattelli R, Scozzafava A, Supuran CT Carbonic anhydrase inhibitors. Part 37. Novel classes of carbonic anhydrase inhibitors and their interaction with the native and cobalt-substituted enzyme: kinetic and spectroscopic investigations. Eur J Med Chem 1996;31:1001–10.

[54] (a) Clare BW, Supuran CT Carbonic anhydrase activators. 3: Structure‐activity correlations for a series of isozyme II activators. J Pharm Sci 1994;83:768–73. 9120804 10.1002/jps.2600830603

[55] (a) Gülçin İ, Scozzafava A, Supuran CT, et al. Rosmarinic acid inhibits some metabolic enzymes including glutathione S-transferase, lactoperoxidase, acetylcholinesterase, butyrylcholinesterase and carbonic anhydrase isoenzymes. J Enzyme Inhib Med Chem 2016;31:1698–702. 26864149 10.3109/14756366.2015.1135914

[56] (a) Syrjänen L, Vermelho AB, Rodrigues Ide A, et al. Cloning, characterization, and inhibition studies of a β-carbonic anhydrase from *Leishmania donovani* chagasi, the protozoan parasite responsible for leishmaniasis. J Med Chem 2013;56:7372–81. 23977960 10.1021/jm400939k

[57] (a) Capasso C, Supuran CT Bacterial, fungal and protozoan carbonic anhydrases as drug targets. Expert Opin Ther Targets 2015;19:1689–704. 26235676 10.1517/14728222.2015.1067685

[58] (a) Vermelho AB, Capaci GR, Rodrigues IA, et al. Carbonic anhydrases from *Trypanosoma* and *Leishmania* as anti-protozoan drug targets. Bioorg Med Chem 2017;25:1543–55. 28161253 10.1016/j.bmc.2017.01.034

[59] (a) D'Ambrosio K, Supuran CT, De Simone G Are Carbonic Anhydrases Suitable Targets to Fight Protozoan Parasitic Diseases?. Curr Med Chem 2018;25:5266–78. 29589529 10.2174/0929867325666180326160121

[60] (a) da Silva Cardoso V, Vermelho AB, Ricci Junior E, et al. Antileishmanial activity of sulphonamide nanoemulsions targeting the β-carbonic anhydrase from *Leishmania* species. J Enzyme Inhib Med Chem 2018;33:850–7. 29708476 10.1080/14756366.2018.1463221 PMC6010131

