# Supplementary material for: Leishmania infantum arginase: biochemical characterization and inhibition by naturally occurring phenolic substances
Source: J Enzyme Inhib Med Chem. 2019 May 24;34(1):1100–9. doi: 10.1080/14756366.2019.1616182 (PMC6534257; doi:10.1080/14756366.2019.1616182)
Supplement: Supplemental Material [file IENZ_A_1616182_SM3036.pdf]

Supplemental Material—*Leishmania infantum* arginase: biochemical characterization and inhibition by naturally occurring phenolic substances

Andreza R. Garcia[1], Danielle M. P. Oliveira[2], Ana Claudia F. Amaral[3], Jéssica B. Jesus[4], Ana Carolina Rennó Sodero[4], Alessandra M. T. Souza[4], Claudiu T. Supuran[5*], Alane B. Vermelho[6], Igor A. Rodrigues[1,7], Anderson S. Pinheiro[2]

[1]Graduate Program in Pharmaceutical Sciences, School of Pharmacy, Federal University of Rio de Janeiro, 21949-900, RJ, Brazil.
[2]Department of Biochemistry, Institute of Chemistry, Federal University of Rio de Janeiro, 21941-909, RJ, Brazil.
[3]Department of Natural Products, Farmanguinhos, FIOCRUZ, 22775-903, RJ, Brazil.
[4]Department of Drugs and Medicines, School of Pharmacy, Federal University of Rio de Janeiro, 21941-590, RJ, Brazil.
[5]NEUROFARBA Department, Università degli Studi di Firenze, Sezione di Scienze Farmaceutiche, Florence, Italy.
[6]Department of General Microbiology, Institute of Microbiology Paulo de Goes, Federal University of Rio de Janeiro, 21941-970, RJ, Brazil.
[7]Department of Natural Products and Food, School of Pharmacy, Federal University of Rio de Janeiro, 21941-902, RJ, Brazil.

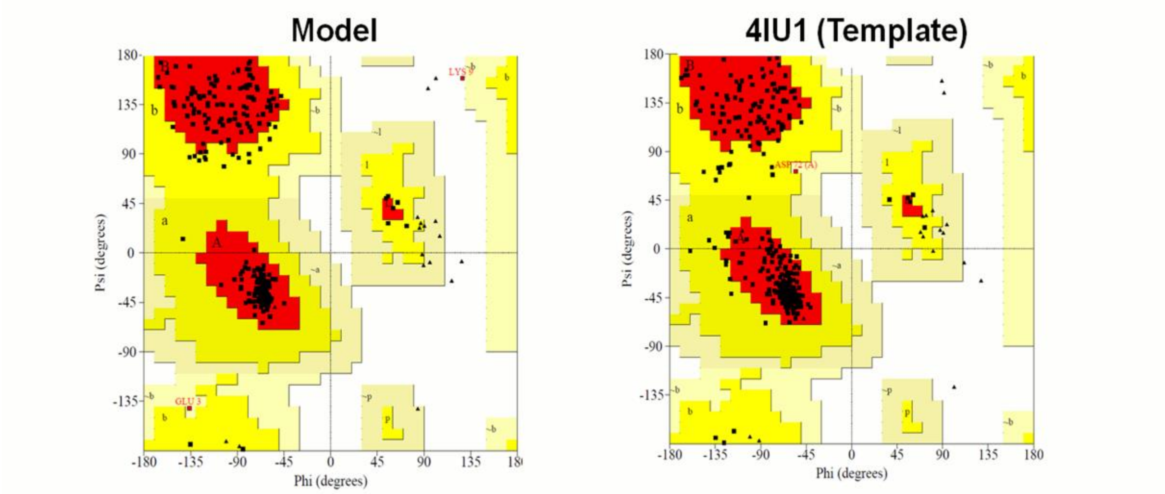

Structures	Regions (%)			
	Most favoured	Allowed	Generously allowed	Disallowed
Model	91.1	8.2	0.4	0.4
4IU1(Template)	87.9	11.7	0.4	0

Supplemental Figure S1: Ramachandran plot of ARGLi three-dimensional model in comparison to the *L. mexicana* arginase (PDB 4IU1) template.

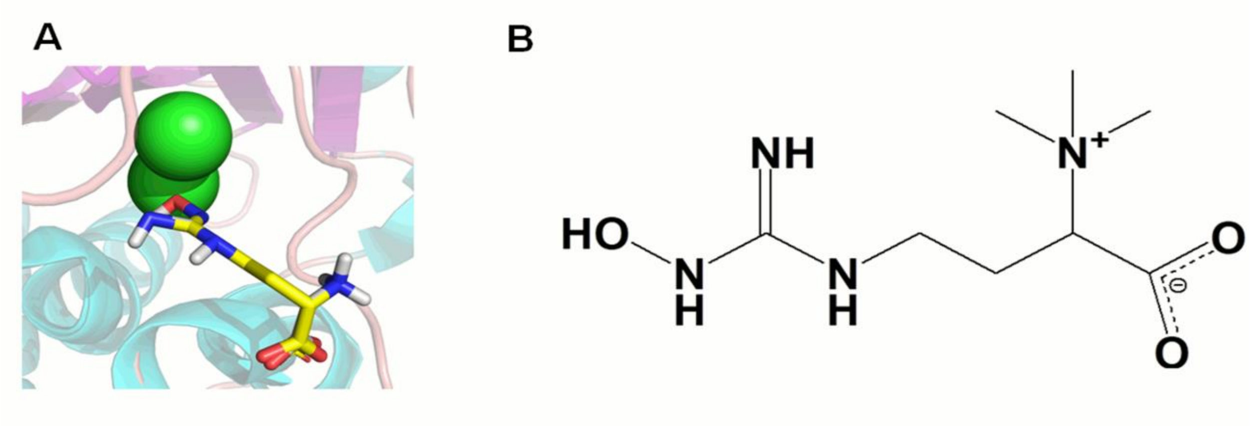

Supplemental Figure S2: (A) 3D structure alignment of the ligand conformation obtained from redocking (white) and the crystallographic structure (yellow). (B) 2D structure of the Nor-N-Omega-Hydroxy-L-Arginine (NNH) inhibitor co-crystallized with *L. mexicana* arginase (PDB 4IU1) used to validate the molecular docking protocol.
